# Oocyte cryopreservation for future fertility: comparison of ovarian response between cancer and non-cancer patients

**DOI:** 10.5935/1518-0557.20190010

**Published:** 2019

**Authors:** Camila Cruz de Moraes, Victoria Furquim Werneck Marinho, Ana Luísa Menezes Campos, Janaína de Souza Guedes, Érica Becker de Sousa Xavier, João Pedro Junqueira Caetano, Ricardo Mello Marinho

**Affiliations:** 1 Pró-Criar Medicina Reprodutiva, Belo Horizonte, MG, Brazil; 2 Faculdade Ciências Médicas Minas Gerais, Belo Horizonte, MG, Brazil; 3 Hospital das Clínicas da Universidade Federal de Minas Gerais, Belo Horizonte, MG, Brazil

**Keywords:** Oocyte cryopreservation, cancer, fertility preservation, oncofertility

## Abstract

**Objective:**

This study aimed to assess whether a diagnosis of cancer interferes with ovarian function prior to the treatment of the disease.

**Methods:**

This observational retrospective study used data from medical records of ovarian stimulation cycles performed for purposes of oocyte cryopreservation.

**Results:**

The included patients had a mean age of 35.13±3.72 years and 51.6% of them were aged between 36 and 40 years. More than half of the patients (57.6%) were single and 82.1% had a normal body mass index (BMI). Most women had not become pregnant (85.5%) or had babies (95.1%) or miscarriages (89.6%) prior to cryopreservation. The mean number of oocytes obtained from non-cancer patients was 11.4±8, while for cancer patients the number was 13.8±9. The mean number of frozen mature oocytes was 9.7±7 for the non-cancer group and 11.2±7.2 for the cancer group. The majority (63.1%) of the patients had up to 10 oocytes frozen per cycle. Breast cancer had the highest incidence among the included patients. There was no significant difference in ovarian response between patients with different types of cancer.

**Conclusion:**

The number of harvested and frozen oocytes from cancer and non-cancer patients indicated that in the two groups response to ovarian stimulation was similar.

## INTRODUCTION

Cryopreservation of human embryos as part of in vitro fertilization (IVF) cycles is a well-established, reliable, routinely performed technique in assisted reproductive technology (ART) laboratories with consistent results and pregnancy rates similar to fresh embryo transfer cycles (Smitz *et al*., 2010; González *et al*., 2012; Donnez & Dolmans, 2015; Jeruss & Woodruff, 2009; Callejo *et al*., 2013). The same procedure has been used to cryopreserve oocytes, but the challenges have been much greater on account of their physical characteristics. Evidence of safety for children born from vitrified oocytes after IVF and standardization of the technique allowed oocyte cryopreservation to no longer be considered experimental (Hammarberg *et al*., 2017; Simoni *et al*., 2017; Noyes *et al*., 2010).

Although it may be used to prevent the development of an excessive number of embryos in ART cycles in reference to patient-related or legal reasons, cryopreservation of oocytes gained importance because it became an option to preserve the fertility of women with cancer and other diseases whose treatment might compromise their ovarian reserve. Additionally, advances in the diagnosis and treatment of cancer achieved in the last four decades have led to significant increases in cure and survival rates and greater appreciation for the quality-of-life of survivors (Lamar & DeCherney, 2009).

However, chemo and radiation therapy used in cancer treatment may compromise future fertility (Smitz *et al*., 2010; González *et al*., 2012; Donnez & Dolmans, 2015; Larsen *et al*., 2003; Carvalho *et al*., 2014; Rodrigues *et al*., 2015; Frydman & Grynberg, 2016) on account of their negative effects on ovarian follicular reserve, in addition to possibly causng infertility or even amenorrhea with hypoestrogenism symptoms (González *et al*., 2012). Oocyte cryopreservation is one of the most widely used strategies to preserve the fertility of cancer patients today, with potentially significant pregnancy rates after thawing and IVF (Alvarez & Ramanathan, 2018).

In recent years, the fastest growing group of individuals seeking oocyte cryopreservation is made up of women with a desire to become mothers, but who are unable to get pregnant at the time they seek care or in the near future for lack of a partner or for professional, economic, or personal reasons. They fear the prospect of having decreased ovarian reserve and fertility over time, particularly as they approach the age of 35 (Espirito Santo *et al*., 2017; Cobo *et al*., 2016).

However, there is a difference between these two groups of women. Patients who seek oocyte cryopreservation to defer maternity for personal reasons are healthy, while cancer patients have a potentially fatal, consumptive condition. For this reason, one might wonder whether cancer patients, even before treatment, might have decreased ovarian reserve, present lower response to ovulation induction, or produce fewer oocytes per cycle for cryopreservation. Despite reports of successful pregnancies following IVF cycles in patients with ovarian malignancies, there is no consensus about the quality or rate of oocyte fertilization when compared to patients who have preserved their oocytes for social reasons (Pal *et al*., 1998; Quinn *et al*., 2017).

Given these uncertainties and the diverging results found in the literature, our study aimed to evaluate ovarian response in oocyte cryopreservation cycles and compare the performance of fertility preservation for cancer patients versus non-cancer patients and find whether the differences may be attributed to the diagnosis of the disease.

## OBJECTIVES

This study aimed to compare the ovarian response of cancer and non-cancer patients in cycles of ovarian stimulation performed for purposes of oocyte cryopreservation based on the number of mature gametes obtained from the two groups of patients.

## MATERIAL AND METHODS

This retrospective study looked into the medical and laboratory records of patients to gather information on ovarian stimulation cycles performed for purposes of oocyte cryopreservation at the Pró-Criar Medicina Reprodutiva clinic from January 2010 to April 2017.

The sample consisted of 187 women, of which 23 (12.3%) underwent oocyte cryopreservation after being diagnosed with cancer. The remainder chose to cryopreserve their oocytes for personal reasons.

The Ethics Committee of the Faculdade Ciências Médicas de Minas Gerais (FCMMG) and Fundação Educacional Lucas Machado (FELUMA) approved the study and assigned it the Certificate of Ethical Presentation no. 60846116.0.0000.5134.

Inclusion criteria: Patients submitted to ovarian stimulation cycles for oocyte cryopreservation due to future cancer treatment and healthy women deferring maternity (self-preservation) seen from January 2010 to April 2017.

Exclusion criteria: Patients with incomplete medical records and infertile patients with cryopreserved oocytes as part of infertility treatment.

The following parameters were analyzed: anthropometric characteristics (age, marital status, and body mass index [BMI]), clinical characteristics, indication for cryopreservation, number of cycles per patient, number of antral follicles, induction protocols, number of gonadotropin ampoules used, day of the cycle in which the puncture was performed, number of harvested oocytes and number of frozen oocytes.

After the descriptive analysis of the groups, they were compared for their characteristics and response to induction.

### Statistical Analysis

Categorical variables were presented as absolute and relative frequencies and numerical variables as mean values ± standard deviation. Numerical variables were submitted to the Shapiro-Wilk normality test. The Wilcoxon Mann-Whitney test was used for independent samples to compare between the mean values of the two groups. The association between categorical variables was assessed using Fisher's exact test or the chi-square test of independence. Statistical analysis was performed on software program R version 3.3.2 and a significance level of 5% was adopted.

### Sample Size Calculation

The sample size was calculated to test the difference between the mean number of oocytes harvested from cancer and non-cancer patients using the following formula (Chow *et al*., 2008):

$$n_{\mathrm{ONC}}=\left(1+\frac{1}{\tau }\right){\left(\sigma \frac{Z_{1-\alpha /2}+Z_{1-\beta }}{d}\right)}^{2},\;e\;\tau =\frac{n_{\mathrm{NON}-\mathrm{ONC}}}{n_{\mathrm{ONC}}}$$

Where σ represents the standard deviation of the number of oocytes harvested in a previous study, z_ (1-α / 2) and z_ (1-β) are quantiles of the normal distribution associated with the significance and power of the test, respectively, and the minimum difference to be detected between the mean oocyte numbers between cancer patients and non-cancer patients. The adoption of a significance level of 5%, a minimum power of 80%, τ=7.13, and the standard deviation of a previous study (Alvarez & Ramanathan, 2018), required that 23 cancer patients and 164 non-cancer patients were included in the study to detect a minimum difference of 5.5 between the mean values of the two groups.

## RESULTS

The mean age of the included women was 35.13±3.72 years, and 51.6% had ages between 36 and 40 years. Cancer patients had a lower mean age (*p*<0.001). The proportion of individuals under 30 years of age was significantly greater among cancer patients.

There was a higher proportion of married women or in steady unions (*p*=0.048). More than half of the patients (57.6%) were single and 82.1% had a normal BMI. Most women had not become pregnant (85.5%) or had babies (95.1%) or miscarriages (89.6%) prior to cryopreservation (Table 1).

**Table 1:** Patient characteristics segregated by group

Variables	Total sample	Non-cancer	Cancer	*p*-value
**n**	187	164	23	
Age*	35.13±3.72	35.72±3.07	30.96±5.14	<0.001^W^
< 30 years	22 (11.8%)	10 (6.1%)	12 (52.1%)	
31 to 35 years	64 (34.4%)	59 (36.2%)	5 (21.7%)	
36 to 40 years	96 (51.6%)	90 (55.2%)	6 (26.1%)	
Over 40 years	4 (2.2%)	4 (2.5%)	-	
**Marital status**				0.048^Q^
Married/steady union	60 (32.6%)	48 (29.8%)	12 (52.2%)	
Divorced, widow*	18 (9.8%)	18 (11.2%)	-	
Single	106 (57.6%)	95 (59%)	11 (47.8%)	
**BMI (kg/m^2^)**	22.69±3.16	22.64±3.24	23.03±2.63	0.481^W^
Low weight	5 (3.7%)	5 (4.3%)	-	
Normal weight	110 (82.1%)	95 (81.2%)	15 (88.2%)	
Pre-obese	15 (11.2%)	13 (11.1%)	2 (11.8%)	
Obese	4 (3%)	4 (3.4%)	-	
**Pregnancies**				
None	148 (85.5%)	130 (86.1%)	130 (86.1%)	
One or two	25 (14.5%)	21 (13.9%)	4 (18.2%)	
**Deliveries**				0.257^F^
None	154 (95.1%)	136 (95.8%)	18 (90%)	
One or two	8 (4.9%)	6 (4.2%)	2 (10%)	
**Miscarriages**				0.476^F^
None	155 (89.6%)	134 (88.7%)	21 (95.5%)	
One or two	18 (10.4%)	17 (11.3%)	1 (4.5%)	

* There was only one widow in the sample The *p*-values refer to the following tests:

Q chi-square of independence,

F Fisher's exact, and

W Wilcoxon Mann-Whitney for independent samples.

BMI - body mass index. The BMI classifications (in kg/m2) were determined as follows (ABESO, 2009): <18.5: low weight; 18.5 to 24.9: normal weight; 25 to 29.9: pre-obese and ≥30: obese

Table 2 shows the number of punctures, harvested oocytes, and frozen oocytes per patient, according to the group to which they belonged (cancer or non-cancer). Most of them (85.9%) had only one follicular puncture.

**Table 2:** Number of cycles, harvested and frozen oocytes per patient for cryopreservation

Variables	Total sample	Non-cancer	Cancer	*p*-value
**N**	187	164	23	
**Follicular punctures**				-
One	158 (85.9%)	138 (85.7%)	20 (87%)	
Two	19 (10.3%)	16 (9.9%)	3 (13%)	
Three or more	7 (3.8%)	7 (4.3%)	-	
**Harvested oocytes**	13.36±9.13	13.04±9.12	15.57±9.11	0.164^W^
Up to 10	83 (45.4%)	75 (46.9%)	8 (34.8%)	
11 to 20	66 (36.1%)	56 (35%)	10 (43.5%)	
21 to 30	23 (12.6%)	21 (13.1%)	2 (8.7%)	
> 30	11 (6%)	8 (5%)	3 (13%)	
**Frozen MII oocytes**	11.27±8.04	11.08±8.17	12.65±7.11	0.174^W^
Up to 10	99 (54.1%	89 (55.6%)	10 (43.5%)	
11 to 20	63 (34.4%)	53 (33.1%	10 (43.5%)	
21 to 30	16 (8.7%)	13 (8.1%)	3 (13%)	
> 30	5 (2.7%)	5 (3.1%)	-	

The *p*-values refer to the following tests:

W Wilcoxon Mann-Whitney for independent samples.

The type of protocol, antagonist, and number of gonadotropin ampoules used according to the cycles performed are shown in Table 3. The antagonist protocol was performed in most of the cycles of the individuals in the non-cancer (74.4%) and cancer (86.4%) groups. For patients in the non-cancer group, the most commonly used ovulatory trigger was hCG, while in the cancer group GnRH agonists were used more often. The mean number of gonadotropin (FSH and hMG) units used was 2,288.1±1,159.4 for non-cancer patients and 2,355.9±1,182 for cancer patients.

**Table 3:** Type of induction protocol, ovulatory trigger, and gonadotropin units used per cycle

Variables	Non-cancer	Cancer	*p*-value
**n**	192	26	
**Induction Protocol**			-
Antagonist	120 (77.4%)	19 (86.4%)	
Long or microflare	26 (16.8%)	1 (4.5%)	
Others	9 (5.8%)	2 (9.1%)	
Ovulation Trigger*			-
hCG	51 (39.8%)	5 (25%)	
Agonist	55 (43%)	15 (75%)	
Ovidrel	22 (17.2%)	-	
**Gonadotropin units**	2.277.5±1.161	2.355.9±1.182	0.827^W^

* Variables with missing data

In the "other" category were included cc + gonad, gonadotropin, Irvine, soft and others The *p*-values refer to the following tests: WWilcoxon Mann- Whitney for independent samples.

W Wilcoxon Mann- Whitney for independent samples.

In 28.3% of the women, follicular puncture was performed after 13 days of induction (Table 4).

**Table 4:** Duration of ovarian stimulation per group

Variables	Total Sample	Non-cancer	Cancer	*p*-value
**N**	218	*192*	26	
Day of puncture*				*-*
9, 10 or 11 days	17 (13.4%)	14 (12.7%)	3 (17.6%)	
12 days	24 (18.9%)	19 (17.3%)	5 (29.4%)	
13 days	36 (28.3%)	32 (29.1%)	4 (23.5%)	
14 days	32 (25.2%)	28 (25.5%)	4 (23.5%)	
15 days	11 (8.7%)	11 (10%)	-	
16,17,26 or 30 days	7 (5.5%)	6 (5.5%)	1 (5.9%)	

* Variables have missing data

Table 5 shows the ovarian response of the patients in the two groups in terms of the number of harvested and frozen oocytes per cycle. There was no significant difference in the ovarian response of the two groups. The mean number of harvested oocytes for non-cancer patients was 11.4±8 *vs.* 13.8±9 for cancer patients. The mean number of frozen mature oocytes was 9.7±7 for the non-cancer group *vs.* 11.2±7.2 for the cancer group. Most (63.1%) of the patients had up to ten oocytes frozen per cycle.

**Table 5:** Number of harvested and frozen oocytes per cycle

Variables	Total sample	Non-cancer	Cancer	*p*-value
**n**	218	192	26	
**Harvested oocytes**		11.4±8	13.8±9	0.185^W^
Up to 10	115 (53.7%)	104 (55.3%)	11 (42.3%)	
11 a 20	71 (33.2%)	61 (32.4%)	10 (38.5%)	
21 a 30	20 (9.3%)	17 (9%)	3 (11.5%)	
> 30	8 (3.7%)	6 (3.2%)	2 (7.7%)	
**Frozen MII oocytes**		9.7±7	11.2±7.2	0.251^W^
Up to 10	135 (63.1%)	122 (64.9%)	13 (50%)	
11 to 20	60 (28%)	50 (26.6%)	10 (38.5%)	
21 to 30	15 (7%)	12 (6.4%)	3 (11.5%)	
> 30	4 (1.9%)	4 (2.1%)	-	

The *p*-values refer to the following test:

W Wilcoxon Mann-Whitney for independent samples

**Graph 1:**
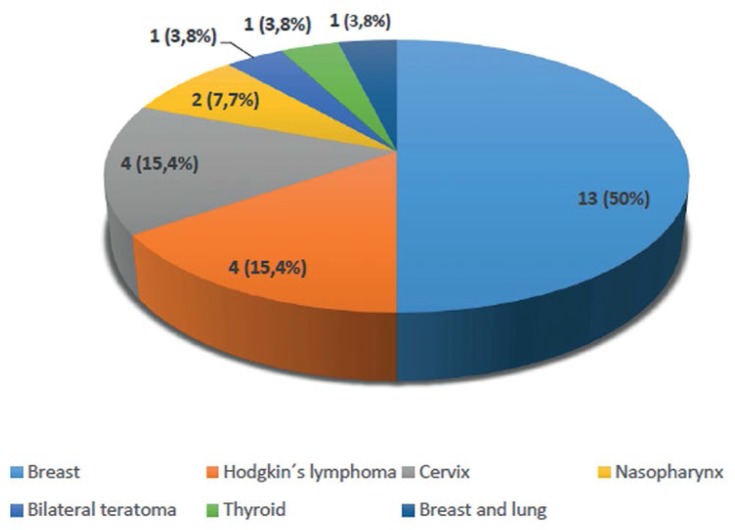
Incidence of cancer types per cycle

Graph 1 shows the incidence of the different cancer types affecting the patients included in the study. Table 6 offers clinical data and information on ovarian response according to each type of cancer.

**Table 6:** Ovarian response according to type of cancer

Variables	Breast	Other	*p*-value
**n**	13	10	
Age (years)	32.46±4.48	29±5.50	0.24 9^W^
BMI (kg/m^2^)	23.50±3.10	22.16±1.19	0.462^W^
Antral follicles	15.36±4.97	17.83±9.43	0.920^W^
Harvested oocytes	14.85±9.49	16.50±9.02	0.534^W^
Frozen oocytes	11.85±7.34	13.70±7.04	0.533^W^

The *p*-values refer to the following test:

W Wilcoxon Mann-Whitney for independent samples

## DISCUSSION

The preservation of female reproductive capacity is a goal that has been pursued for a long time by women and specialists in Reproductive Medicine. The decrease of the ovarian reserve with age, followed by its exhaustion in menopause, has kept many women from realizing the dream of becoming mothers. A specific group of individuals has taken a special interest in preserving fertility: young women diagnosed with cancer and yet with good chances of surviving, whose treatment - chemotherapy, radiation therapy, surgery - may strongly compromise their chances of becoming pregnant in the future (Cobo *et al.,* 2016).

The choice of a less aggressive therapy for the gonads should be attempted, but it is not always possible. The use of drugs such as GnRH analogs for ovarian protection during chemotherapy has arguable efficacy (Bliss *et al*., 2010) and the freezing of ovarian tissue, despite its track record of about one hundred births, is still considered experimental (Rodriguez‐Wallberg *et al*., 2016).

On the other hand, in recent years mature oocyte (MII) cryopreservation has become an established option, with well-defined protocols and good outcomes. The development of the vitrification technique allowed oocyte cryopreservation to become a safe and effective technique, and an extremely attractive option for individuals wishing to preserve fertility (Noyes *et al*., 2010; Cobo *et al*., 2016; Garcia-Velasco *et al*., 2013).

In addition to cancer patients, women who need to postpone the possibility of gestation on account of benign diseases or personal plans have also started to pursue this alternative, drastically increasing the demand for these techniques (Cobo *etal*., 2016).

Several recent studies have reported pregnancies resulting from the transfer of embryos from frozen oocytes at levels similar to fertilization cycles using fresh oocytes. Most of these studies were performed with oocytes donated by young women or healthy infertile women, who did not have cancer (Noyes *et al*., 2010; Nagy *et al*., 2009).

Although not many studies have confirmed a relationship between cancer and decreased or impaired ovarian function, it is known that during tumor development several immunosuppressive molecules are released from cancer cells, as well as toxic substances that contribute to the establishment of a tumor immunosuppressive environment (Nishida & Kudo, 2017).

The quality of the oocytes of cancer patients can only be assessed in terms of their correlation with pregnancies and births if compared to a control group. However, these studies are difficult to organize since few cancer survivors have tried to conceive with frozen eggs.

We may, however, indirectly assess the ovarian reserve through the response to ovarian stimulation performed to harvest oocytes for cryopreservation, and by then comparing it to the findings of a control group. The ovarian reserve represents the reproductive potential of the ovaries, and relates to the number and quality of the remaining oocytes. A good way to measure this reserve is by counting antral follicles and measuring the ovarian volume by ultrasound examination. This measurement, performed up to the third day of the menstrual cycle, has been correlated with the ovarian response to induction with gonadotropins and indirectly related to the ovarian reserve (ASRM, 2015).

In our study, the number of mature (MII) oocytes frozen per cycle was used to compare the ovarian reserve of cancer and non-cancer patients. The control group included healthy women who had their oocytes cryopreserved for personal reasons. We did not include patients who had oocytes frozen as part of infertility treatment, since these individuals may have impaired ovarian reserve or response. Patients seeking oocyte cryopreservation for personal reasons were deemed adequate controls because they were healthy and potentially fertile.

After analyzing our data and considering the number of IVF cycles performed at the clinic and the potential of young patients diagnosed with cancer, we found that the number of cancer patients that had their oocytes frozen is still small. Many are the reasons for this finding, a reality present in almost any country. They revolve primarily around the lack of information among physicians and patients, the troubles with establishing links between oncologists and specialists in reproductive medicine to promptly initiate treatment, and (evidently) the costs involved. Therefore, we advocate the need for a multidisciplinary approach for these patients, enforced through improved communication between all areas involved in order to avoid unnecessary delays in the evaluation and treatment of patients. Although ovarian stimulation takes some time, randomly started cycles may produce mature oocytes in up to 14 days in the vast majority of the cases, as in our sample (Letourneau *et al*., 2017; Vaiarelli *et al*., 2017; Pereira *et al*., 2017). The associated costs are also an issue, and ideally the government and health insurances should reimburse clinics for the procedure, as it happens in European countries.

Cancer patients were slightly younger than the patients who had their oocytes cryopreserved for social reasons (Table 1). Cancer patients had a mean age of 30.9 years and most (52.1%) had ages ranging between 19 and 30 years, while non-cancer patients had a mean age of 35.7 years. Patients seeking to postpone motherhood tend to have their oocytes cryopreserved around the age of 35, when they realize they will not conceive soon. Cancer patients, however, seek help at the time of diagnosis. This group tends to be younger, since older women may have already had the children they wanted before they were diagnosed with cancer (Bleyer & Barr, 2009).

In theory, the fact that the patients in this group were younger may have affected the number of harvested and frozen oocytes. However, controls were also relatively young, with a mean age of 35 years, and little difference has been observed between the IVF outcomes of individuals aged 30 and 35. Therefore, we realized that the impact of the age difference would be small.

The group of patients who had their oocytes cryopreserved for social reasons consisted mainly of single women. This data point reflects the concern these women had with the decrease they will experience in their reproductive capacity over the years and their attempt to increase the chances of becoming pregnant in the future. The contemporary sociocultural environment undoubtedly leads women to seek economic, professional, and personal stability before forming a family. As described by some authors, the troubles of finding the right partner and the lack of commitment of couples to forming a family are two of the main reasons for delaying maternity (Cobo *et al*., 2016; Hodes-Wertz *et al*., 2013).

As observed in other studies, breast cancer was the most frequent diagnosis in the cancer group (Alvarez & Ramanathan, 2018). Something that attracted our attention was the fact that individuals with breast cancer are usually older than other patients, although the age difference is not statistically significant.

The results also showed that there was no significant difference between the two groups of patients (non-cancer and cancer) in relation to the number of cycles performed. The vast majority (85.9%) had only one follicular puncture (Table 2). We expected a greater number of punctures in the non-cancer group, since these patients theoretically have more time to repeat induction procedures to ensure a greater number of oocytes for future fertilization. On the other hand, the small number of cancer patients submitted to more than one cycle may be explained by the short time they had until the start of cancer treatment. They must be rapidly referred to an ART center to initiate hormonal induction, undergo follicular puncture, and have their oocytes frozen. The choice of the antagonist protocol for the vast majority (86.4%) of the cancer patients is justified by the fact that it is a shorter protocol, with less time until the start of stimulation, thus minimizing the time to the initiation of cancer treatment (Nishida & Kudo, 2017). The recent use of random start and double stimulation protocols may increase the possibility of performing more than one cycle within a shorter period of time, with a large number of oocytes being harvested to increase the chances of future pregnancy (Kim *et al*., 2015). It is unclear what the ideal number of oocytes might be to ensure pregnancy, but a recent study estimated that it might take ten to 15 oocytes for patients up to 35 years of age to reach a plateau of birth probability of 85.2% (Cobo *et al*., 2016). Most (63.1%) of our patients had up to ten MII oocytes frozen, indicating a 40.8% probability of birth according to Cobo *et al*. (2016), considering that our patients were aged 35 or younger. Since the cancer group had mostly patients aged 30 or younger, this statistic finding applies very well to our study. On the other hand, the non-cancer group had ages ranging between 36 and 40 years, thus dropping the probability of birth to 25.8% when eight to ten oocytes are frozen. To reach a plateau of 35.6% of probability of birth, the individuals in this group would require 11 MII oocytes on average.

Although this was not our main endpoint, we noticed that the antral follicle counts before induction were not different between the two groups (59.1% had between 10 and 20 antral follicles), suggesting that cancer had no effect in the ovarian reserve of the two groups (Table 7) (ASRM, 2015).

**Table 7:** Number of antral follicles per cycle

Variables	Total sample	Non-cancer	Cancer	*p*-value
**N**	218	192	26	
Antral follicles*				0.341^Q^
< 10	23 (18.1%)	22 (20%)	1 (5.9%)	
10 to 20	75 (59.1%)	64 (58.2%)	11 (64.7%)	
> 20	29 (22.8%)	24 (21.8%)	5 (29.4%)	

The *p*-values refer to the following tests:

Q chi-square of independence

Our main objective was to assess the ovarian response to induction, measured by the number of harvested and frozen oocytes. There was no difference in the number of oocytes harvested per cycle (Table 5), with the non-cancer group having a mean of 11.4 oocytes and the cancer group 13.8 oocytes harvested. The mean number of frozen MII oocytes for the non-cancer and cancer groups was 9.7 and 11.2, respectively. Most patients had up to 10 oocytes in each cycle in both groups (Cardozo *et al*., 2015; Nurudeen *et al*., 2016). A promising finding was that the number of cryopreserved oocytes was not significantly different between the cancer and non-cancer patients. Since cryopreserved oocytes are deemed mature and with good microscopic quality, they may potentially be fertilized by intracytoplasmic sperm injection (ICSI) and form embryos to be transferred and possibly generate pregnancies.

The data from our study supported existing studies in that cancer did not significantly impact the ovarian reserve or the response to stimulation, since most patients seeking to preserve fertility prior to cancer treatment do not have a history of infertility. With appropriate counseling and multidisciplinary care, patients diagnosed with early-stage cancer may have levels of ovarian response to hormonal induction similar to cancer-free individuals of similar ages (González *et al*., 2012; Alvarez & Ramanathan, 2018; Cardozo *et al*., 2015; Nurudeen *et al*., 2016).

Some authors found different results. Alvarez & Ramanathan reported that patients with hematological or breast cancer had more MII oocytes than patients with gynecological cancer. Pal *et al.* also described negative impacts on the quality and behavior of oocytes of cancer versus control groups, with significant decreases in the proportion of harvested mature oocytes and lower fertilization rates in cancer patients compared to controls (Alvarez & Ramanathan, 2018; Pal *et al*., 1998). Perhaps the lack of negative impacts on ovarian response seen in our cancer patients stemmed from the fact that they did not have advanced stage disease.

Some authors tried to segregate patient ovarian response based on the type of cancer they had, in an attempt to find whether different forms of the disease might have differentially affected ovarian response. Alvarez & Ramanathan reported that patients with gynecological cancer had fewer MII oocytes harvested than individuals with breast or hematologic cancer (Alvarez & Ramanathan, 2018).

In our series, cancer patients were divided into two groups: one featuring individuals with breast cancer and another with patients with other types of cancer. We found no significant difference in the ages, BMI, number of antral follicles, or number of harvested and frozen oocytes between the two groups. The small number of cancer patients in our sample may have affected our findings.

We believe that the greatest limitation of our study was the small number of cancer patients enrolled. More studies should be carried out in partnership with other centers so that larger volumes of data are analyzed and the results better represent what occurs with this population of women.

## CONCLUSION

Our study found similar levels of response to ovarian stimulation with cancer and non-cancer patients, since the number of harvested and frozen oocytes in the two groups was similar.

Cancer patients with good prognosis and whose treatment may compromise fertility may be offered ovarian stimulation and egg collection for cryopreservation, to thus improve their chances of becoming pregnant in the future with the aid of established assisted reproductive technology treatments.

The growing number of individuals seeking oocyte cryopreservation for social reasons deserves equal attention. Advanced maternal age translates into increased risk of not having children. In addition, more effective ART treatments offer better outcomes for younger patients, who respond better to medications and are less likely to have aneuploid oocytes.

## References

[r1] ABESO - Diretrizes Brasileiras de Obesidade (2009). Associação Brasileira para o Estudo da Obesidade e da
Síndrome Metabólica.

[r2] Alvarez R, Ramanathan P (2018). Fertility preservation in female oncology patients: the influence
of the type of cancer on ovarian stimulation response. Hum Reprod.

[r3] ASRM - Practice Committee of the American Society for Reproductive
Medicine (2015). Testing and interpreting measures of ovarian reserve: a committee
opinion. Fertil Steril.

[r4] Bleyer A, Barr R (2009). Cancer in young adults 20 to 39 years of age:
overview. Semin Oncol.

[r5] Bliss SP, Navratil AM, Xie J, Roberson MS (2010). GnRH signaling, the gonadotrope and endocrine control of
fertility. Front Neuroendocrinol.

[r6] Callejo J, Salvador C, González-Nuñez S, Almeida L, Rodriguez L, Marqués L, Valls A, Lailla JM (2013). Live birth in a woman without ovaries after autograft of
frozen-thawed ovarian tissue combined with growth factors. J Ovarian Res.

[r7] Cardozo ER, Thomson AP, Karmon AE, Dickinson KA, Wright DL, Sabatini ME (2015). Ovarian stimulation and in-vitro fertilization outcomes of cancer
patients undergoing fertility preservation compared to age matched controls:
a 17-year experience. J Assist Reprod Genet.

[r8] Carvalho BR, Rodrigues JK, Marinho RM, Caetano JPJ, Rosa e Silva ACJS (2014). Visão geral sobre preservação da fertilidade
feminina depois do câncer. Reprod Clim.

[r9] Chow S, Shao J, Wang H (2008). Sample Size Calculations in Clinical Research.

[r10] Cobo A, García-Velasco A, Coello A, Domingo J, Pellicer A, Remohí J (2016). Oocyte vitrification as an efficient option for elective
fertility preservation. Fertil Steril.

[r11] Donnez J, Dolmans MM (2015). Ovarian tissue freezing: current status. Curr Opin Obstet Gynecol.

[r12] Espirito Santo EV, Dieamant F, Petersen CG, Mauri AL, Vagnini LD, Renzi A, Zamara C, Oliveira J, Baruffi RLR, Franco Jr JG (2017). Social oocyte cyopreservation: a portrayal of Brazilian
women. JBRA Assist Reprod.

[r13] Frydman R, Grynberg M (2016). Introduction: Female fertility preservation: innovations and
questions. Fertil Steril.

[r14] Garcia-Velasco JA, Domingo J, Cobo A, Martínez M, Carmona L, Pellicer A (2013). Five years' experience using oocyte vitrification to preserve
fertility for medical and nonmedical indications. Fertil Steril.

[r15] González C, Boada M, Devesa M, Veiga A (2012). Concise review: fertility preservation: an update. Stem Cells Transl Med.

[r16] Hammarberg K, Kirkman M, Pritchard N, Hickey M, Peate M, McBain J, Agresta F, Bayly C, Fisher J (2017). Reproductive experiences of women who cryopreserved oocytes for
non-medical reasons. Hum Reprod.

[r17] Hodes-Wertz B, Druckenmiller S, Smith M, Noyes N (2013). What do reproductive-age women who undergo oocyte
cryopreservation think about the process as a means to preserve
fertility?. Fertil Steril.

[r18] Jeruss JS, Woodruff TK (2009). Preservation of fertility in patients with cancer. N Engl J Med.

[r19] Kim JH, Kim SK, Lee HJ, Lee JR, Jee BC, Suh CS, Kim SH (2015). Efficacy of random-start controlled ovarian stimulation in cancer
patients. J Korean Med Sci.

[r20] Lamar CA, DeCherney AH (2009). Fertility preservation: state of the science and future research
directions. Fertil Steril.

[r21] Larsen EC, Müller J, Schmiegelow K, Rechnitzer C, Andersen AN (2003). Reduced ovarian function in long-term survivors of radiation- and
chemotherapy-treated childhood cancer. J Clin Endocrinol Metab.

[r22] Letourneau JM, Sinha N, Wald K, Harris E, Quinn M, Imbar T, Mok-Lin E, Chien AJ, Rosen M (2017). Random start ovarian stimulation for fertility preservation
appears unlikely to delay initiation of neoadjuvant chemotherapy for breast
cancer. Hum Reprod.

[r23] Nagy ZP, Chang CC, Shapiro DB, Bernal DP, Elsner CW, Mitchell-Leef D, Toledo AA, Kort HI (2009). Clinical evaluation of the efficiency of an oocyte donation
program using egg cryo-banking. Fertil Steril.

[r24] Nishida N, Kudo M (2017). Oncogenic Signal and Tumor Microenvironment in Hepatocellular
Carcinoma. Oncology.

[r25] Noyes N, Labella PA, Grifo J, Knopman JM (2010). Oocyte cryopreservation: a feasible fertility preservation option
for reproductive age cancer survivors. J Assist Reprod Genet.

[r26] Nurudeen SK, Douglas NC, Mahany EL, Sauer MV, Choi JM (2016). Fertility Preservation Decisions Among Newly Diagnosed Oncology
Patients: A Single-Center Experience. Am J Clin Oncol.

[r27] Pal L, Leykin L, Schifren JL, Isaacson KB, Chang YC, Nikruil N, Chen Z, Toth TL (1998). Malignancy may adversely influence the quality and behaviour of
oocytes. Hum Reprod.

[r28] Pereira N, Voskuilen-Gonzalez A, Hancock K, Lekovich JP, Schattman GL, Rosenwaks Z (2017). Random-start ovarian stimulation in women desiring elective
cryopreservation of oocytes. Reprod Biomed Online.

[r29] Quinn MM, Cakmak H, Letourneau JM, Cedars MI, Rosen MP (2017). Response to ovarian stimulation is not impacted by a breast
cancer diagnosis. Hum Reprod.

[r30] Rodrigues JK, Campos JR, Marinho RM, Xu J, Zelinski MB, Stouffer RL, Marinho RM, Silva ACJS, Caetano JPJ, Rodrigues JK (2015). Desenvolvimento folicular e maturação
oocitária in vitro. Preservação da fertilidade: Uma Nova Fronteira em Medicina
Reprodutiva e Oncologia.

[r31] Rodriguez-Wallberg KA, Tanbo T, Tinkanen H, Thurin-Kjellberg A, Nedstrand E, Kitlinski ML, Macklon KT, Ernst E, Fedder J, Tiitinen A, Morin-Papunen L, Einarsson S, Jokimaa V, Hippeläinen M, Lood M, Gudmundsson J, Olofsson JI, Andersen C (2016). Ovarian tissue cryopreservation and transplantation among
alternatives for fertility preservation in the Nordic countries -
compilation of 20 years of multicenter experience. Acta Obstet Gynecol Scand.

[r32] Simoni MK, Mu L, Collins SC (2017). Women's career priority is associated with attitudes towards
family planning and ethical acceptance of reproductive
technologies. Hum Reprod.

[r33] Smitz J, Dolmans MM, Donnez J, Fortune JE, Hovatta O, Jewgenow K, Picton HM, Plancha C, Shea LD, Stouffer RL, Telfer EE, Woodruff TK, Zelinski MB (2010). Current achievements and future research directions in ovarian
tissue culture, in vitro follicle development and transplantation:
implications for fertility preservation. Hum Reprod Update.

[r34] Vaiarelli A, Venturella R, Vizziello D, Bulletti F, Ubaldi FM (2017). Dual ovarian stimulation and random start in assisted
reproductive technologies: from ovarian biology to clinical
application. Curr Opin Obstet Gynecol.

